# A country-wide teledermatoscopy service in Estonia shows results comparable to those in experimental settings in management plan development and diagnostic accuracy: A retrospective database study

**DOI:** 10.1016/j.jdin.2023.02.019

**Published:** 2023-04-06

**Authors:** Christian Koop, Priit Kruus, Riina Hallik, Hannes Lehemets, Elen Vettus, Marianne Niin, Peeter Ross, Külli Kingo

**Affiliations:** a Dermtest OÜ, Tallinn, Estonia; b Department of Health Technologies, Tallinn University of Technology, School of Information Technology, Tallinn, Estonia; c East Tallinn Central Hospital, Clinic of Internal Medicine, Centre of Oncology, Tallinn, Estonia; d Dermato-oncology Clinic OÜ, Tallinn, Estonia; e Department of Dermatology and Venerology, Faculty of Medicine, Institute of Clinical Medicine, University of Tartu, Tartu, Estonia; f Tartu University Hospital, Dermatology Clinic, Tartu, Estonia; g East Tallinn Central Hospital, Tallinn, Estonia

**Keywords:** diagnostic accuracy, melanoma, management plan accuracy, nevus, skin cancer, screening, teledermoscopy, teledermatology, teledermatoscopy

## Abstract

**Background:**

Teledermatoscopy accuracy has been examined in experimental settings and is recommended for primary care despite lacking real-world implementation evidence. A teledermatoscopy service has been provided in Estonia since 2013, where lesions are evaluated based on the patient’s or general practitioner’s suggestion.

**Objective:**

The management plan and diagnostic accuracy of a real-world store-and-forward teledermatoscopy service for melanoma diagnosis were evaluated.

**Methods:**

A retrospective study analyzed 4748 cases from 3403 patients using the service between October 16, 2017 and August 30, 2019 by matching country-wide databases. Management plan accuracy was calculated as the percentage of melanoma found that was managed correctly. Diagnostic accuracy parameters were sensitivity, specificity, and positive and negative predictive values.

**Results:**

Management plan accuracy for melanoma detection was 95.5% (95% CI, 77.2-99.9). Diagnostic accuracy showed a sensitivity of 90.48% (95% CI, 69.62-98.83) and a specificity of 92.57% (95% CI, 91.79-93.31).

**Limitations:**

Matching the lesions was limited to SNOMED CT location standard precision. Diagnostic accuracy was calculated based on a combination of diagnosis and management plan data.

**Conclusion:**

Teledermatoscopy for detecting and managing melanoma in real-world clinical practice displays results comparable with those in experimental setting studies.

**Capsule Summary**

• Teledermatoscopy is well examined in experimental setting studies with satisfactory management plan and diagnostic accuracy results.
• Teledermatoscopy implemented in everyday dermatologic care showed comparable management plan and diagnostic accuracy. This gives further confidence that practicing clinicians can use its advantages safely to improve regular dermatologic care for patients.

## Introduction

Early detection and treatment of skin cancer are essential to improve prognosis.^1^, ^2^, ^3^, ^4^ Teledermatology helps to enable earlier assessment and treatment.^5^ Professional organizations suggest teledermatoscopy for pigmented lesions for everyday care,^6^ although most research relies on experimental setting studies where the service is provided for a limited time and to a limited patient population in specially controlled environments.^7^, ^8^, ^9^, ^10^, ^11^, ^12^, ^13^ Financially self-sustainable teledermatoscopy services have been evaluated^14^; however, research on melanoma management plan and diagnostic accuracy in these regular clinical practice settings is lacking because of methodological difficulties, such as the need for patient follow-up or excision of all suspicious lesions for histopathology.^15^ Here, a different approach was taken using Estonia's long-established country-wide national health information system (NHIS), which was matched with a country-wide teledermatoscopy service database (TDDB). Active since February 8, 2013, the service had helped to evaluate 11,658 cases and had been used by 103 different general practitioners (GPs) and 11 different dermatologists by August 30, 2019.

The study aimed to evaluate the management plan accuracy of a financially self-sustaining store-and-forward teledermatoscopy service for melanoma diagnosis at the primary care level. Because the working diagnosis is not regularly provided as part of the management plan, especially when melanoma is suspected, diagnostic accuracy was calculated based on a combination of diagnosis and management plan data.

### Hypotheses

The authors hypothesized that all melanomas (malignant melanoma of the skin - C43 and melanoma *in situ* - D03) would be correctly managed by dermatologists via the teledermatoscopy service.

The authors hypothesized that the diagnostic accuracy of teledermatoscopy would be similar in real-world and experimental settings.^7^, ^8^, ^9^, ^10^, ^11^^,^^13^

## Methods

This retrospective 2-database study was conducted in accordance with the World Health Organization Declaration of Helsinki, the International Society for Pharmacoeconomics and Outcomes Research (ISPOR) checklist for retrospective studies^16^ and the Standards for the Reporting of Diagnostic Accuracy Studies (STARD) criteria.^17^ Approvals were granted by the Ethics Committee for Human Research of the Estonian Institute for Health Development (ruling 393) and the Estonian Bioethics and Human Research Council of the Ministry of Social Affairs of Estonia (ruling 1.1-12/2531). The full research protocol can be requested from the corresponding author.

### Index test

The index test was defined as the teledermatoscopy examination that resulted in a management plan (“Excision,” “Biopsy,” “Visit dermatologist,” “Checkup nevus in 1, 3, 4, 5, 6, or 12 months,” and “No further action needed”) and a dermatologist diagnosis provided as an International Classification of Diseases (ICD-10) code. The management plan and diagnosis were provided based on dermatoscopic and macroscopic images of the lesion taken at the GP office by a doctor or nurse with an iPhone 6 or higher using a Dermlite DL1 smartphone add-on dermatoscope. The data were retrieved from the TDDB, which also includes lesion-specific information on location and size as well as patients’ clinical and baseline demographic data.

### Reference test

A positive reference test was defined as a melanoma histopathology diagnosis obtained from the NHIS, including information on the location, time, and date of the lesion’s excision. The pathologist analyzing the histology specimen had no access to the photographs of the lesion. A negative reference test was defined as the absence of a melanoma histopathology diagnosis within 1.5 years after the dermatologist’s diagnosis.

The SNOMED CT browser Estonian version,^18^ the NIH ICD conversion program,^19^ and the World Health Organization International ICD-10 browser^20^ were used to convert SNOMED CT to ICD-10 coding. For statistical analysis, SPSS 28 and MedCalc Statistical Software^21^ were used.

### Service process and participants

Teledermatoscopy was demanded either by patients or recommended by GPs. The request for evaluation, including patient and lesion anamnesis and images, was sent by a GP to a dermatologist. Management plan and diagnosis were provided approximately within 2 days and reported to the patient by the GP. If indicated, they referred the patients for an excision, which then resulted in the creation of a histopathology diagnosis entry in the NHIS.

All patients who used the service between October 16, 2017 and August 30, 2019 (5389 cases) were included in the study. The final sample consisted of 4748 cases from 3403 patients. Each case was considered independent to determine diagnostic accuracy.^8^ The minimum required sample size of 2930 cases for a maximum 95% CI width of 10% was calculated based on the method by Buderer assuming a melanoma prevalence in the sample of 1.85%,^12^ sensitivity of 0.83, and a specificity of 0.92 for the detection of melanoma.^22^, ^23^, ^24^

### Analysis

#### Matching

The index and reference tests were matched if they met the following criteria: (1) the latter was performed within 1.5 years (= 548 days) after the former, (2) they had the same patient ID, and (3) similar location of lesions. Missing or inconclusive data on 1 of the 3 criteria led to an exclusion of the patient before matching.

Generally, if multiple consecutive index tests concerned the same lesion, the later ones were excluded. Only if the first index test contained a management plan recommendation for a checkup and could not be matched with a positive reference test, the first index tests were excluded.

If multiple positive reference tests could be matched with the same index test, the 2 with the least time passed in between, were matched.

If there were still several options to match the index with reference tests, after the rules set out above were applied, then the matching was conducted twice, assuming the worst- and the best-case scenario, in which the primary priority was to achieve either the lowest or the highest number of false-negative cases possible, respectively. For readability purposes, the study presents only the worst-case scenario.

After the matching, inconclusive index tests were excluded along with reference tests without matched index tests. The definition of inconclusive differs between management plan and diagnostic accuracy calculation (see below).

#### Management plan accuracy

The management plan accuracy was defined as the percentage of any melanomas found by histopathology that were managed correctly with an “excision” or “biopsy”^25^^,^^26^ or a “visit dermatologist.” A management plan to checkup in 1 month was considered inconclusive and excluded from the calculation; all other management plans were considered incorrect.

#### Diagnostic accuracy

For diagnostic accuracy calculations, an index test was considered positive, if the diagnosis was melanoma (C43/D03) or if the diagnosis was neoplasm of uncertain or unknown behavior (D48.5 and D48) and the management plan was “excision” or “biopsy.” Valid inconclusive index tests were those recommending “visit dermatologist” or “checkup nevus in 1 month.” Invalid inconclusive were index tests, in which images were deemed unusable by the dermatologist. Both valid and invalid inconclusive index tests were excluded from binary statistics but reported and considered for the test yield.^27^^,^^28^ All other combinations of diagnoses and management plans were considered negative.

## Results

### Participants and cases

**Fig 1:**
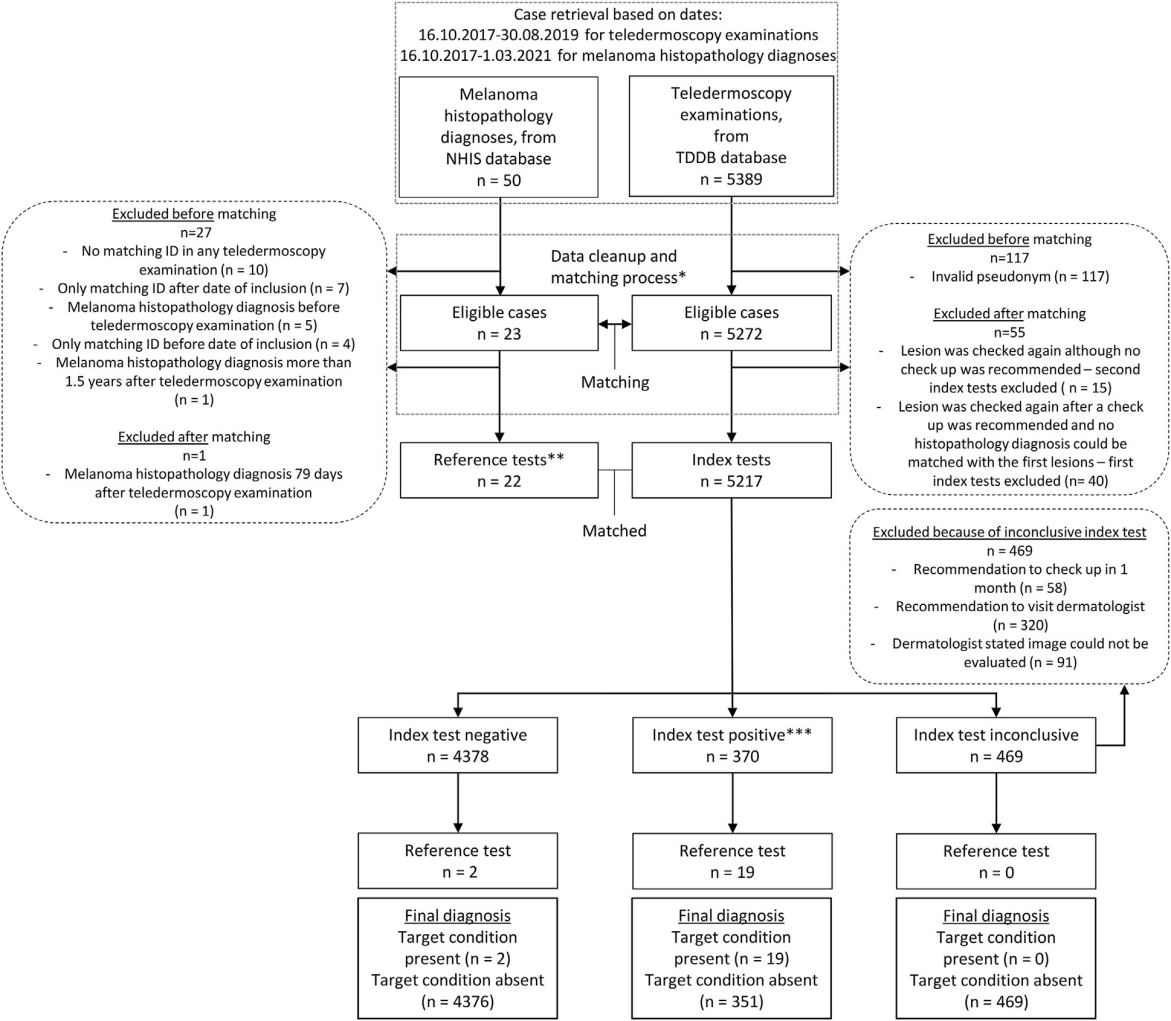
STARD flow diagram expanded for case inclusion from NHIS and TDDB databases and matching. ∗Case matching process steps were as follows: 1. Data cleanup before matching process – cases contained valid information on patient ID, date, and nevus location. Additionally, the melanoma histopathology diagnosis was performed within 1.5 years (= 548 days) after the index test and cases had the same patient ID. 2. Matching process – cases were matched if the locations of the lesions matched. 3. Exclusion after the matching **–** inconclusive index tests and duplicate diagnoses were excluded. ∗∗The reference test was histopathology. If no histopathology diagnosis could be found in the NHIS within 1.5 years after the index test (teledermatoscopy examination), the reference test was considered negative. ∗∗∗The index test was positive when the diagnosis was C43/D03 or D48.5/D48 with the recommendation to perform “excision” or “biopsy.”

Data retrieval from TDDB produced 5389 potentially eligible index tests. Of these, 5272 contained data for matching criteria (1), (2), and (3), which allowed matching with 23 positive reference tests. Of these 23, one was excluded after matching because it could be matched with the same index test as another similar positive reference test that was performed earlier. Another positive reference test was matched with a valid inconclusive index test in which the management plan recommended visiting a dermatologist and was thus only eligible for management plan accuracy calculation. Therefore, 22 positive reference tests were included for the calculation of management plan accuracy and 21 for the calculation of diagnostic accuracy. Next, 469 index tests were excluded because of 3 different reasons for inconclusiveness, leaving a total of 4748 cases for analysis. Specifics of matching, inclusion process, and exclusion reasons can be seen in Fig 1 and Table I.

**Table I:** Overview of melanoma histopathology diagnoses matched with teledermatoscopy examinations

Patient	Index test (teledermatoscopy examination)			Positive reference test (melanoma histopathology diagnosis)			Days between	
	ICD-10 diagnosis	Recommendation of action by dermatologist	Location	SNOMED CT Identifiers	ICD-10 diagnosis	Location	Diagnosis and excision	Diagnosis and histopathology
1	C43	Surgery and histopathology	Torso front	55320002‡	C43	Entire skin of abdomen	7	10
2	D22	Surgery and histopathology	Torso front	55320002‡	C43	Entire skin of abdomen	19	23
3	D48.5	Surgery and histopathology	Torso front	77986002§	D03	Entire skin of abdomen	43	53
4	D48.5	Surgery and histopathology	Torso front	2092003‖	C43	Entire skin of chest	19	174
5	C43	Surgery and histopathology	Torso back	2092003	C43	Entire skin of back	10	17
6	C43	Surgery and histopathology	Torso back	55320002‡	C43	Entire skin of back	11	21
7	D22	Follow-up in 12 mo	Torso back	77986002§	D03	Entire skin of back	86	99
8	D48.5	Surgery and histopathology	Torso back	55320002‡	C43	Entire skin of back	25	77
9	D48.5	Surgery and histopathology	Torso back	2092003‖	C43	Entire skin of back	7	40
10	D48.5	Surgery and histopathology	Torso back	61217001¶	D03	Entire skin of back	8	18
11	D48.5	Surgery and histopathology	Torso back	77986002§	D03	Entire skin of back	61	133
12	C43	Surgery and histopathology	Torso back	2092003‖	C43	Skin tissue	2	21
13	D48.5	Surgery and histopathology	Right arm left side	77986002§	D03	Entire skin of shoulder	20	29
14	D48.5	Surgery and histopathology	Head and neck front	55320002‡	C43	Entire skin of cheek	12	15
15	D48.5	Surgery and histopathology	Head and neck front	2092003‖	C43	Entire skin of cheek	22	29
15∗	-	-	-	2092003‖	C43	Entire skin of cheek	72	79
16	D48.5	Surgery and histopathology	Head and neck front	55320002‡	C43	Entire skin of chin	11	18
17	C43	Surgery and histopathology	Head and neck back	55320002‡	C43	Entire skin of head	12	19
18	D48.5	Surgery and histopathology	Left leg front	55320002‡	C43	Entire skin of foot	53	61
19	D48.5	Surgery and histopathology	Left leg front	2092003‖	C43	Entire skin of lower leg	9	24
20†	D48.5	Dermatologist consultation	Left arm back	77986002§	D03	Entire skin of forearm	14	32
21	D48.5	Surgery and histopathology	Left arm back	44474009#	C43	Entire skin of upper extremity	50	58
22	D48.5	Surgery and histopathology	Left arm up	55320002‡	C43	Entire skin of upper arm	469	476

∗ Excluded from management plan and diagnostic accuracy calculation because the potential index test match could already be matched with a similar positive reference test that was performed earlier.

† Excluded from diagnostic accuracy calculation because a dermatologist consultation was recommended.

‡ 55320002 Superficial spreading melanoma (morphologic abnormality).

§ 77986002 Melanoma *in situ* (morphologic abnormality).

‖ 2092003 Malignant melanoma, no International Classification of Diseases for Oncology subtype (morphologic abnormality).

¶ 61217001 Hutchinson melanotic freckle (morphologic abnormality).

# 44474009 Malignant melanoma in Hutchinson melanotic freckle (morphologic abnormality).

### Test results

Of the 22 matched diagnoses, 16 were C43 and 6 were D03. Table I shows the overview of melanoma histopathology diagnoses matched with teledermatoscopy examinations and their inclusion in the management plan and diagnostic accuracy calculations.

The cross-tabulation is displayed in Table II. The test yield was 92.6% (*n* = 4748 cases divided by all cases *n*= 5389 minus invalid cases *n* = 172 and minus invalid inconclusive results *n* = 91). Management plan and diagnostic accuracy calculations are shown in Table III.

**Table II:** Cross-tabulation of the teledermatoscopy examination as index test results vs the results of histopathology diagnosis during a 1.5 years observation period as reference tests

		Histopathology diagnosis		Total
		Negative	Positive	
Dermatologist’s diagnosis	Negative	4376	2	4378
	Positive	351	19	370
Total		4727	21	4748

**Table III:** Management plan and diagnostic accuracy calculations for melanoma detection by teledermatoscopy

*N* = 4748	Management accuracy∗װ	Sensitivity∗§	Specificity∗§	PPV†‡§	NPV†‡§
%	95.5	90.48	92.57	5.14	99.95
(95% CI)	(77.2-99.9)	(69.62-98.83)	(91.79-93.31)	(4.36-6.04)	(99.83-99.99)

*PPV*, Positive predictive value; *NPV*, negative predictive value.

∗ CIs are exact Clopper Pearson CI.

† CI are standard logit CI.^29^

‡ The prevalence of melanoma in the sample was 0.44 % (95% CI, 0.27-0.68).

§ Diagnostic accuracy was calculated based on diagnosis and management plan data.

װ Only concerns the management of histopathologically confirmed melanoma (*N* = 22).

The mean age of participants counted per case was 39, with an SD of 17.706, range of 0 to 93. Women were predominantly using the service (*n* = 3126), which is 65.8% of all cases. Additional characteristics referring to the risk of developing melanoma are shown in Supplementary Table 1 (available via Mendeley at https://data.mendeley.com/datasets/4tp6grcvb7/1). Based on the ICD-10 codes, the most prevalent nonmelanoma diagnosis was melanocytic nevi-D22 in 2816 cases (59.3%). The second largest group was seborrheic keratosis-L82, concerning 1049 (22.1%) cases, and the third was neoplasm of uncertain or unknown behavior-D48 with 332 (8.3%) cases. Other notable diagnoses were hemangioma and lymphangioma-D18 with 167 (3.5%) cases, benign neoplasms of skin-D23 with 128 (2.7%), other disorders of pigmentation-L81 with 53 (1.1%), and benign neoplasms of connective and other soft tissue-D21 with 42 (0.9%) cases. The rest of the diagnoses represented <1% of the total count. Dermatologists recommended no further action in 2776 (58.5%) cases, checkups in 1177 (24.8%), and excision and histopathology examination or biopsy in 795 (16.7%) cases. Lesions were located on the trunk/back (*n* = 1274; 26.8%), trunk/abdomen and chest (*n* =1177; 24.8%), head/neck (*n* = 958; 20.2%), leg/foot (*n* = 714; 15.0%), hand/arm (*n* = 421; 8.9%). and trunk/sides (*n* =204; 4.3%).

From the dermatologist‘s diagnosis, a mean of 45.52 days (SD = 99.45; range, 2-469 days) passed until the excision and a mean of 67.38 days (SD = 102.90; range, 10-476 days) to the histopathology diagnosis.

## Discussion

### Management plan and diagnostic accuracy

Comparable studies reported management plan accuracy as 95.83% to 100% (1 out of 24 melanomas was misdiagnosed as squamous cell carcinoma),^7^ 100% (1 out of 48 melanomas was misdiagnosed as solar lentigo but recommended for monitoring),^9^ 93.75% (of 16 total melanomas, 2 melanomas were diagnosed as activated melanocytic nevus and junctional nevus but excised, 1 melanoma was diagnosed as a multiforme reaction),^10^ 100 % (5 melanoma correctly managed),^11^ and 91.3% (2 out of 23 melanomas potentially mismanaged).^13^ Although the reference standard for benign lesions was a face-to-face clinical examination, only 2 studies implemented a follow-up plan to confirm nonmalignancy.^9^^,^^11^ Hence, melanomas might have been overseen. This study has comparable results with 95.5% (95% CI, 77.2-99.9; 1 melanoma mismanaged out of 22). The potential mismatch of a D03 histopathology diagnosis with a D22 teledermatoscopy diagnosis and a 12-month checkup recommendation was considered mismanagement.

Comparable studies examining the diagnostic accuracy of teledermatoscopy regarding melanoma with experimental settings are reported with sensitivities, specificities, and *n* of 71%, 95%, and 256^7^; 96%, 71%, and 128^9^; 81%, 93% and 45^10^; 100%, 95% and 235^11^; and 74%, 94% and 600,^13^ respectively. The sensitivity of 90.48% (95% CI, 69.62-98.83) and specificity of 92.57% (95% CI, 91.79-93.31) of this study can be considered comparable results. The 2 potential misdiagnoses (a D03 and a C43 histopathology diagnosis matched with a D22 dermatologist’s diagnosis from a teledermatoscopy examination each) are plausible matches regarding the times between the diagnoses: 86 and 19 days, respectively.

There was only one incident where 2 histopathology diagnoses were registered for one patient (see lesion no. 15 in Table I). This suggests that lesion-directed screening performs similarly to total body examination.^30^

### Aspects of study design

The statistical prevalence of confirmed melanoma in the sample (0.44%) is similar to that in population-based screening programs^30^^,^^31^ and smaller than in prospective studies (eg, 9.38%,^7^ 37.5%,^9^ 35.6%,^10^ 2.13%,^11^ and 3.8%.^13^) In those studies, medical personnel (eg, GPs) (pre) selected the patients who were suspected of having malignant lesions, but in the teledermatoscopy service under evaluation, mostly the patients themselves initiated the teledermatoscopy examination. While prospective studies need a higher prevalence for economic reasons, for this study a natural prevalence sufficed owing to the high sample size of 4748 (5389 before exclusions) recruited during everyday health care provision.

The retrospective study design posed challenges. One limitation was matching the lesion locations because histopathology diagnoses had low SNOMED CT location standard precision (eg, “entire skin of back”), unlike the TDDB that allowed selecting lesion locations by finger tapping on the smartphone screen. The problem of documenting anatomic body surface locations is generally known in the field of dermatology and is currently at the center of scientific debate.^32^ As a result of the low precision, there was ambiguity concerning the matching of teledermatoscopy examinations with positive histopathology examinations in 2 cases. The authors took a conservative stance and highlighted only the results of the worst-case scenario, although the best-case scenario was equally plausible. In the equally likely best-case scenario of management plan accuracy, the recommendation to checkup the one mismanaged melanoma (see lesion no. 7 in Table I) in 6 months was considered mismanagement but might have helped to detect this *in situ* lesion earlier than the 12 months checkup recommendation in the worst-case scenario. In the equally likely best-case scenario of diagnostic accuracy, 1 of 2 misdiagnosed melanomas (see lesion no. 2 in Table II) was diagnosed as a D03 with a management plan to excise and thus would have improved the diagnostic accuracy, leaving only one misdiagnosed melanoma.

A diagnosis-reporting protocol cannot be established in studies relying on retrospective data. Because it was common practice for dermatologists not to give melanoma (C43) or melanoma *in situ* (D03) diagnoses without histopathology confirmation, they often diagnosed a neoplasm of uncertain or unknown behavior: skin (D48.5 or D48) and recommended excision or biopsy. Thus, those cases had to be considered as positive index test results, possibly inflating the sensitivity. This also greatly limits the comparability with other studies of the diagnostic accuracy of teledermatoscopy.

Because the diagnostic accuracy of dermatoscopy depends on the level of the examiner’s experience,^33^, ^34^, ^35^ it might have differed based on the examiners’ various expertise levels in this study and was potentially decreased. The same is true for the persons taking the images. This disadvantage must be considered when comparing the results with those studies with more experimental settings employing only expert dermatologists and experts taking the images.

A major strength of the study was the 1.5 years observation period, which was enabled by the country-wide database NHIS that archived all information on histopathology reports in the examined time frame. If a rapidly growing melanoma was missed, it would likely have caused clinical symptoms in 1.5 years and led to its excision or biopsy.^3^ Then, a histopathology report in the NHIS would have been created and a false-negative case could have been detected. Although Wang et al^25^ considered a 1-year period to be a sufficient time for missed melanomas to be detected, slow-growing melanoma, however, might be missed by this approach because they quadruple in the area only in 3.5 years.^36^ A future follow-up research based on the same data sources would enable to capture of a longer time period and further leverage the opportunity of having a country-wide database of histopathologic reports for analysis.

## Conclusion

Teledermatoscopy implemented in a regular health care setting as a screening test for melanoma detection shows management plan and diagnostic accuracy comparable with that of teledermatoscopy examined in experimental setting studies.

## Conflicts of interest

Dr Koop, Kruus, Hallik, Lehemets, Dr Ross, and Dr Kingo have declared financial interest in Dermtest OÜ, the company providing the software, which enables teledermatoscopy in Estonia. Drs Vettus, and Niin have no conflicts of interest to declare.
